# Study on the Tribological Performance of Copper-Based Powder Metallurgical Friction Materials with Cu-Coated or Uncoated Graphite Particles as Lubricants

**DOI:** 10.3390/ma11102016

**Published:** 2018-10-18

**Authors:** Xin Zhang, Yongzhen Zhang, Sanming Du, Zhenghai Yang, Tiantian He, Zhen Li

**Affiliations:** 1 Wuhan Research Institute of Materials Protection, China Academy of Machinery Science and Technology, Wuhan 430030, China; zhangxin_sdha@163.com; 2 National United Engineering Laboratory for Advanced Bearing Tribology, Henan University of Science and Technology, Luoyang 471023, China; dsming@haust.edu.cn (S.D.); yzh772029@haust.edu.cn (Z.Y.); tthe@haust.edu.cn (T.H.); lizhen@haust.edu.cn (Z.L.)

**Keywords:** powder metallurgical material, graphite, friction coefficient, friction temperature, friction film

## Abstract

The tribological performance of copper-based powder metallurgical material is much influenced by the interfacial bonding between the components and matrix. By adding Cu-coated or uncoated graphite particles as a lubricant, two types of copper-based powder metallurgical materials were prepared via spark plasma sintering (SPS). The hardness, relative density, and thermal conductivity of the two specimens were firstly measured. Using an inertial braking test bench and temperature measuring instrument, the average friction coefficients, instantaneous friction coefficients, and friction temperatures of the two specimens were tested under different test conditions, and the wear rates were calculated accordingly. Based on the analysis of surface morphologies and elements distribution after the tests, the mechanisms of wear and formation of friction films were discussed. The results show that with the lubricant of Cu-coated graphite, the hardness, relative density, thermal conductivity, and interfacial bonding between the graphite and matrix can be greatly improved. Under the same test condition, the average friction coefficient, wear rate, and friction temperature of the specimen with added Cu-coated graphite are both lower than those of the specimen with added uncoated graphite. The two specimens show different variation trends in the instantaneous friction coefficient during the tests, and the variation of the instantaneous friction coefficient at a high initial test speed is also different from that at a low initial test speed for each specimen. The two specimens also show differences in the continuity of friction film and the content of graphite and oxide in the friction film.

## 1. Introduction

The requirements for friction materials in high-speed braking applications are strict. They demand that the friction materials can provide not only a high and stable friction coefficient, but also good wear resistance during braking [1,2,3]. Among various candidates, copper-based powder metallurgical friction materials are widely used in braking pads owing to their excellent mechanical property and thermal conductivity [4,5]. The friction and wear property of copper-based metallurgical friction materials are influenced by many factors, such as the composition, preparation process, service conditions, and so on [6,7,8,9,10]. Among those factors, the material composition has a strong impact. As it is known, solid lubricants are commonly used in powder metallurgical materials and usually play an important role in stabilizing friction and reducing wear. Due to its low friction coefficient, good thermal conductivity, and lower costs, graphite has been widely used in the materials [11,12,13,14]. However, the poor interfacial bonding between graphite and the metal matrix can induce the inferior matrix continuity and mechanical property of sintered materials. This would also cause the easy removal of graphite on the surface, thus reducing the lubrication effect and increasing wear [14,15].

For copper-based powder metallurgical materials, it is an effective measure to enhance the interfacial bonding between the graphite and matrix in sintering by using metal-coated graphite [13,14,15,16,17]. The enhancement of interfacial bonding can induce the variation of material property, which has been studied by many researchers. Using Cu-coated graphite, Park et al. [15] found that the pristine graphite changed to graphite oxide (GO) on the interface during sintering, and the interface was quite continuous without an obvious gap on it. Additionally, the composite exhibited excellent thermal conductivity and relative density. Moustafa et al. [12] studied the friction and wear property of a copper-graphite composite with three contents (8%, 15%, and 20%) of Cu-coated or uncoated graphite. The results showed that the mechanical property and relative density of the Cu-coated graphite composite were higher than those of an uncoated graphite composite at the same content, and the friction coefficient and wear rate of the Cu-coated graphite composite were both lower than those of the uncoated graphite composite under the same condition. They also found that the two composites had the same wear mechanisms, and the deformation capacity influenced the surface state and wear. In the study of the dry-sliding tribological property of a bronze-graphite composite with Ni-coated (11.7%, 15%, and 18.3%) or uncoated graphite (4.5%) carried out by Cui et al. [13], the friction coefficients and wear rates of three Ni-coated graphite composites were all lower than those of an uncoated graphite composite at the same test condition. Moreover, the graphite-rich mechanical mixed layers of three Ni-coated graphite composites were all continuous. After studying the microstructures and friction properties of bronze-graphite composites, Zhao et al. [14] concluded that the bonding quality between the matrix and graphite could be improved by using Cu-coated or Ni-coated graphite, and the friction coefficient of the Ni-coated graphite composite was more stable than that of the Cu-coated and uncoated graphite composite at the same sliding time. Compared with the uncoated graphite composite, the Cu-coated and Ni-coated graphite composites showed a higher integrity of friction films. The results of the above studies are valuable and helpful for other researchers to carry out further investigations on the influences of metal-coating of graphite on the tribological property of materials. As it is known, friction temperature has a strong impact on the formation of a friction film on the surface during friction, further affecting the friction coefficient [18,19,20,21,22]. Under the same conditions, the enhancement of interfacial bonding between the graphite and matrix by using metal-coated graphite can greatly influence the friction temperature. However, in previous studies, under the two cases of adding metal-coated and uncoated graphite, the dynamic variation characteristics of the friction coefficient and temperature, as well as their correlation, were seldom reported, and the formation mechanisms of friction film have not been studied in depth.

In this work, two types of copper-based powder metallurgical materials were prepared with Cu-coated and uncoated graphite. The hardness, relative density, and thermal conductivity of the two specimens were measured and compared. In the friction and wear tests under different braking conditions, the differences between the two specimens in the average friction coefficient and wear rate were studied. By measuring the temperatures near the friction surface and instantaneous friction coefficients during the braking processes of different conditions, the variation characteristics of friction temperature and friction coefficient with test time and the relationship between them for the two specimens were also discussed. Based on the analysis of microstructures, surface morphologies, elements distribution, and phases composition after friction tests, the interfacial bonding, formation mechanisms of friction film, and wear mechanisms of the two specimens were explored.

## 2. Experiments

### 2.1. Materials

**Figure 1:**
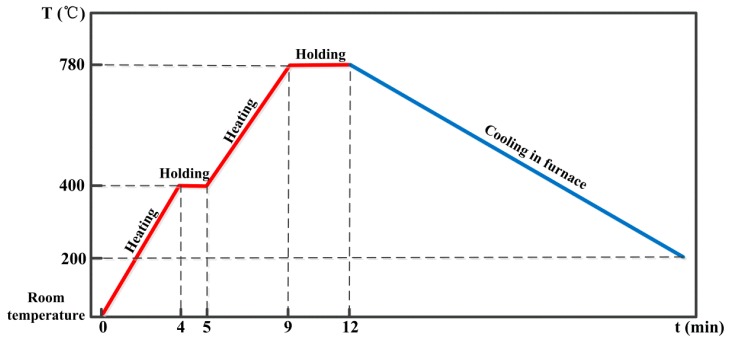
Sintering process route.

**Figure 2:**
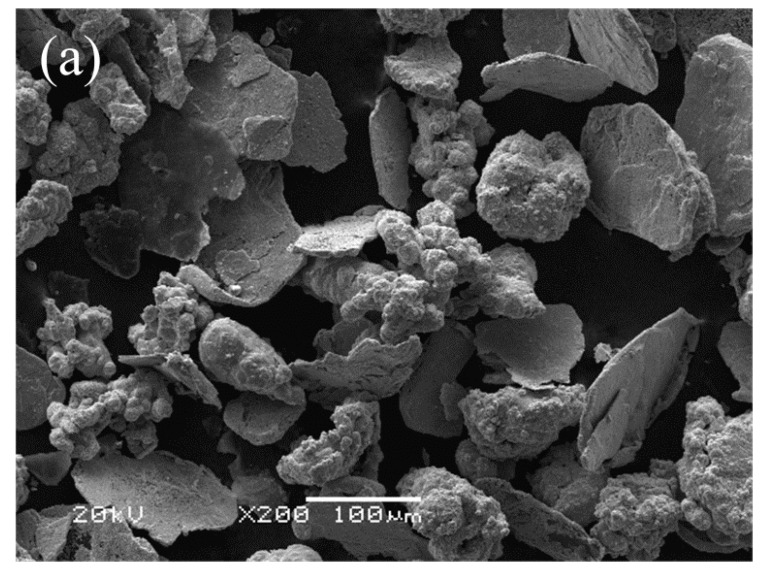

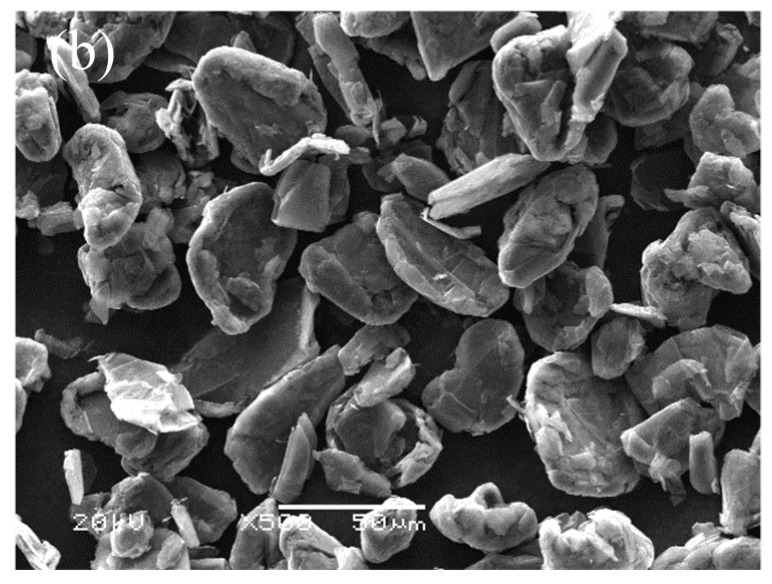
SEM photographs of Cu-coated graphite (**a**) and uncoated graphite (**b**).

Two types of friction specimens were prepared via SPS, and the sintering temperature and pressure were 780 °C and 30 MPa, respectively. The sintering process is shown in Figure 1. For the two specimens, the matrix member chosen was copper, the matrix strengthening members were iron and tin, and the friction member was silicon dioxide [23,24]. For the lubricant, either Cu-coated graphite or uncoated graphite (Hengxin Co., Hangzhou, China) was used in the two specimens. The SEM photographs of the two types of graphite particles are shown in Figure 2, where it is seen that the graphite particles were effectively covered by Cu-coating (Figure 2a). The average sizes of copper, iron, tin, silicon dioxide, Cu-coated graphite, and uncoated graphite particles were 75, 25, 75, 6.5, 75, and 50 μm, respectively. The chemical compositions of the two specimens (denoted as specimen S1 and S2) are shown in Table 1. The Cu-coating on graphite was carried out by using the electroless coating method, and the mass ratio of copper to graphite was 1/1, so the graphite contents in the two specimens were both 4%. Cylindrical pad samples with a diameter of 20 mm and thickness of 15 mm were prepared by sintering. Brinell hardness tester (320HBS-3000) (Fangyuan Co., Jinan, China) was used to measure the hardness of the specimens at the load of 250 kg and the indentation ball was 5 mm in diameter. Using Archimedes method, the density of the two specimens was measured, followed by the calculation of relative density. Thermal conductivity coefficients of the two specimens were tested by using a laser thermal conductivity testing instrument (LFA-1000) (Linseis Co., Selb, Germany) with a test temperature of 600 °C and reference material of graphite.

### 2.2. Friction and Wear Test

**Figure 3:**
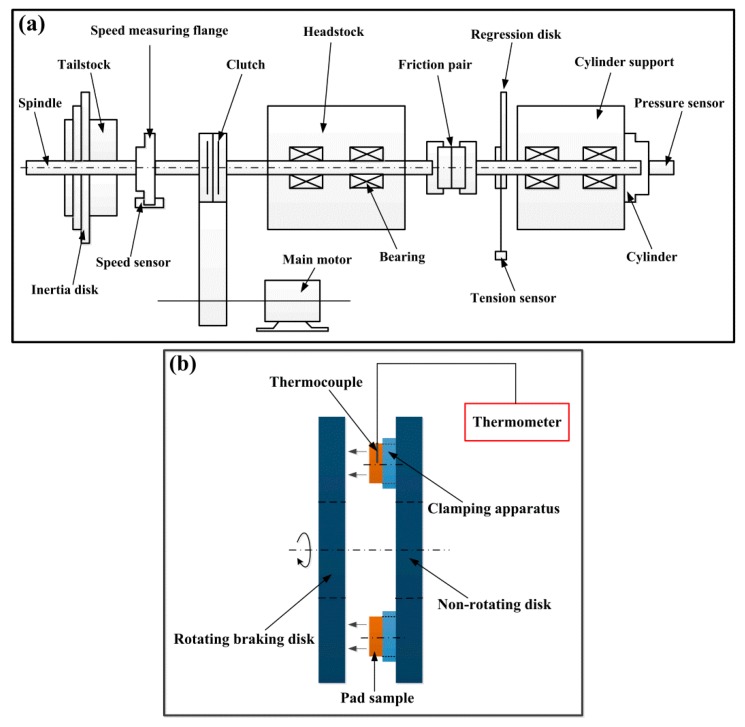
Schematic of test bench structure (**a**) and friction pair (**b**).

The friction and wear properties of the two specimens were tested on an inertial braking test bench (MM 1000-II) (Shuntong Co., Xi’an, China). The braking disk (25Cr2MoVA steel) with a diameter of 160 mm and thickness of 9 mm was chosen. The hardness and surface roughness (R_a_) of the disk were 37 HRC and 1.0 µm, respectively. The structure of the test bench and the friction pair are shown schematically in Figure 3. The test inertia was 0.5 kg m^2^ after computation (Equation (A1)). Test groups are shown in Table 2. For the two specimens, the test pressures (p) applied were 0.4 and 0.8 MPa, and the initial test speeds (v_0_) were 100, 150, 200, and 250 km/h. During the tests, the instantaneous friction torque, friction speed, and pressure were measured by the sensors, and the instantaneous friction coefficient could be calculated by the computer equipped on the test bench (Equation (A2)) accordingly. For each group, the test was repeated five times, the average friction coefficient and braking time of each test were recorded, and the material loss per unit of energy consumed (wear rate, mg/kJ) was computed (Equation (A3)). For the test groups 1, 4, 5, and 8 for specimen S1 and groups 9, 12, 13, and 16 for specimen S2, the temperature near the friction surface was also measured to study its variation during the tests. The measurement position was 1.5 mm beneath the friction surface. An electronic thermometer (TES1384) (Taishi Co., Taipei, China) with a memory function and a K-type thermocouple were used in the tests. The data collection interval was set as 2 s. Meanwhile, the instantaneous power input during the test process was also computed (Equation (A4)).

### 2.3. Materials Analysis and Characterization

**Figure 4:**
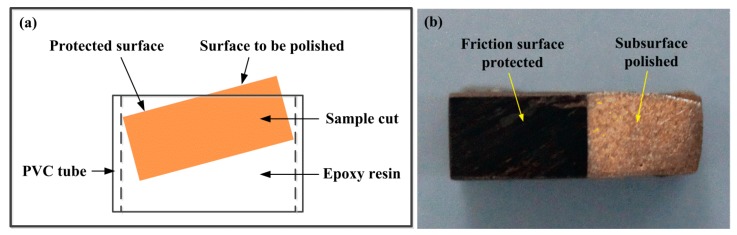
Schematic of inlaid sample (**a**) and inclined-plane sample (**b**).

In order to study the differences in the microstructure and wear mechanism of the two specimens, the sintered specimens and friction surfaces of different test conditions (groups 1, 4, 5, and 8 for specimen S1 and groups 9, 12, 13, and 16 for specimen S2) were analyzed and compared using SEM and x-ray diffraction (XRD) patterns. For better comparing the surface morphologies and wear mechanisms of the two specimens, several cylindrical samples were shaped to inclined-plane form, as shown in Figure 4, which is also helpful for a direct analysis of the differences between the surface and subsurface in elements distribution. In the preparing of the inclined-plane sample, part of the friction surface was protected by using epoxy resin, and another part unprotected needed to be polished. After polishing was finished, the samples were immersed in acetone to remove the resin. Then, the morphology and elements distribution of the surface and subsurface were analyzed by SEM/EDS.

## 3. Results and Discussion

### 3.1. Materials Feature

The hardness, relative density, and thermal conductivity coefficient of the two specimens are given in Table 1. It is evident that the hardness, relative density, and thermal conductivity coefficient of specimen S1 are all higher than those of specimen S2. This is attributed to the fact that the addition of Cu-coated graphite in specimen S1 is helpful to strengthen the interfacial bonding between the graphite and copper matrix, thus leading to better matrix continuity in specimen S1 than specimen S2. Therefore, specimen S1 possesses a high mechanical property and thermal conductivity [12,13,14,17]. This would be beneficial for reducing the wear and thermal damage of materials during friction.

**Figure 5:**
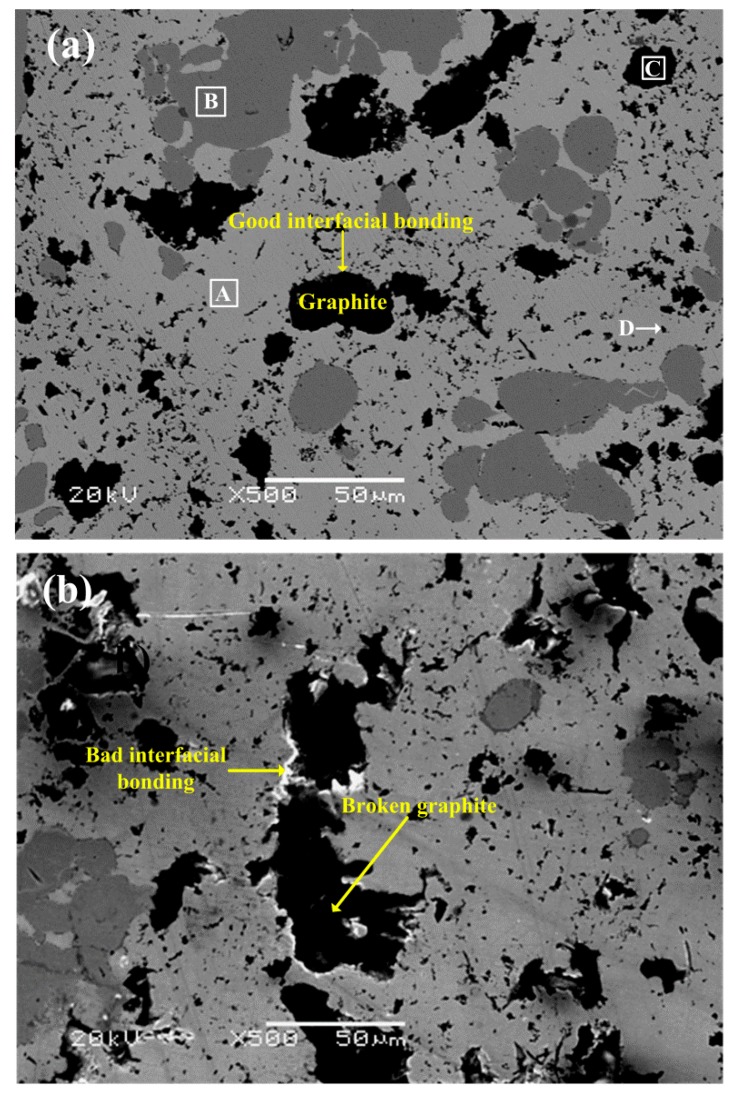
Microstructures of specimen S1 (**a**) and S2 (**b**).

Figure 5 shows the microstructures of the two specimens prepared, and Table 3 shows the EDS analysis results of area A, B, C, and D in Figure 5a. As shown in Figure 5a, it is seen that the matrix of specimen S1 mainly consists of Cu and Cu-Sn solid solution, such as area A. As shown by the EDS results for area B and C, the large grey and black particles are Fe and graphite, respectively. The dispersed dark particles are SiO_2_, as indicated by the white arrow. The components distribution of specimen S2 (Figure 5b) is the same as that of specimen S1. However, it is evident that the interfacial bonding between the graphite and matrix of specimen S2 is quite weak, causing some graphite particles to be detached from the matrix and broken easily when subjected to external forces [13,14,19,25]. The poor interfacial bonding greatly reduced the matrix continuity. Such cases are not found in specimen S1, indicating a better interfacial bonding between the graphite and matrix of specimen S1 than that of specimen S2.

### 3.2. Average Friction Coefficient and Wear Rate

The friction and wear test results are shown in Table 2. It is evident that the braking times of the two specimens are both shortened with the increase of test pressure at the same initial test speed. At the same pressure and speed, the braking time of specimen S2 is slightly shorter than that of specimen S1. The maximum friction temperatures of the two specimens elevate with the increase of speed and pressure, and the maximum friction temperature of specimen S1 is much lower than that of specimen S2 at the same condition.

**Figure 6:**
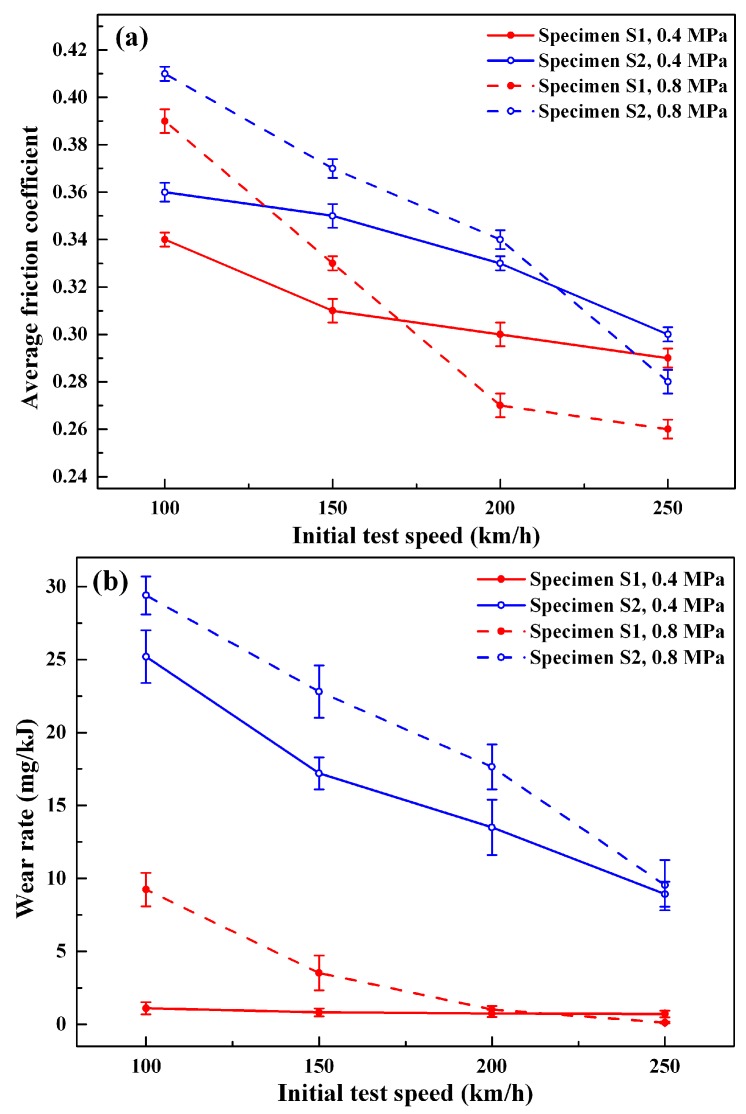
Average friction coefficient (**a**) and wear rate (**b**) under different conditions.

The variations of average friction coefficient and wear rate of the two specimens with the initial test speed at two different pressures are shown in Figure 6. For specimen S1 and S2, at the same pressure, the average friction coefficients all decrease with the increase of speed (Figure 6a). This is because the elevating friction temperature caused by increasing speed can induce the decrease of the surface mechanical property, even surface softening; meanwhile, the capacity of deformation of the surface can be strengthened. These changes are helpful to the formation of surface friction film, such as transfer film and oxide film, and so on [12,25]. Therefore, the average friction coefficients decrease. For the two specimens, when the speed is below 200 and 250 km/h for specimen S1 and S2, respectively, the friction coefficients under high pressure are higher than those under low pressure at the same speeds. However, when the speeds increase, the friction coefficients of high pressure are lower than those of low pressure. By comparing the friction coefficients of the two specimens, it is found that the friction coefficients of specimen S1 are all lower than those of specimen S2 at the same pressure and speed, which is beneficial to reducing material wear, as shown in Figure 6b. Therefore, it can be deduced that the enhancement of interfacial bonding and matrix continuity by the addition of Cu-coated graphite is helpful to improve not only the mechanical property, but also the wear resistance, of material, consistent with the results by Moustafa [12] and Zhao et al. [14]. From Figure 6b, it is also seen that the wear rates of specimen S1 at 0.8 MPa and specimen S2 at 0.4 and 0.8 MPa show the same variation feature with speed, but for specimen S1 at 0.4 MPa, the wear rate shows slight variation with speed.

### 3.3. Instantaneous Friction Coefficient and Friction Temperature

**Figure 7:**
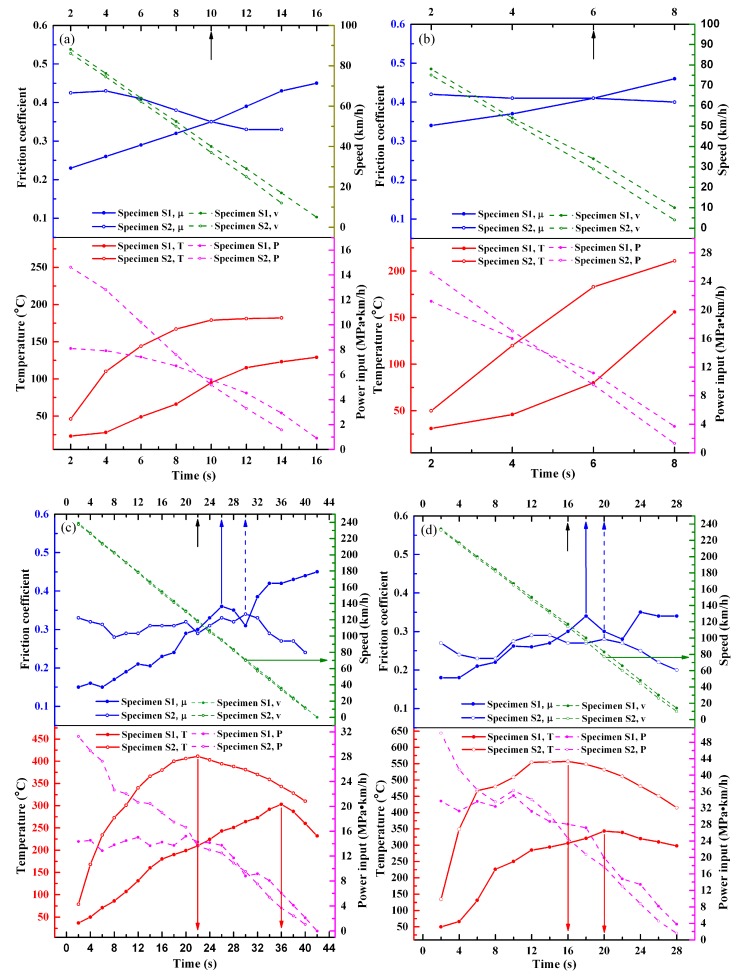
The variation of friction temperature (T), instantaneous friction coefficient (μ), speed (v), and power input (P) during the braking process under different test conditions: (**a**) 100 km/h, 0.4 MPa; (**b**) 100 km/h, 0.8 MPa; (**c**) 250 km/h, 0.4 MPa; (**d**) 250 km/h, 0.8 MPa.

Figure 7 shows the variations of friction speed, friction coefficient, power input, and friction temperature of the two specimens with test time. It is seen that the instantaneous friction coefficients, power inputs, and friction temperatures of the two specimens show different varying characteristics with time at the same condition. At the four test conditions shown in Figure 7, the variations of speed with time all show the feature of an almost linear decrease; that is, the four test processes can be treated as constant deceleration processes, which is necessary for friction materials to be used in braking. Furthermore, under the four conditions, the difference between specimen S1 and S2 in friction speed is small at the same test time, especially at high speed, so the difference between them in the power input is mainly influenced by the friction coefficient at the same test time.

Due to the differences between the two specimens in the friction coefficient and thermal conductivity, the power inputs and friction temperatures of them show different variation features during test processes. It is evident that both the temperature rising rate of the beginning stage and the temperature value at the same test time of specimen S1 are lower than those of specimen S2 under the same condition. For each condition, before the friction process reaches a certain time (10, 6, 22, and 16 s indicated by the black arrow in Figure 7a–d, respectively), at the same test time, the friction coefficient of specimen S2 is higher than that of specimen S1, so the power input of specimen S2 is also higher. In addition, the thermal conductivity of specimen S2 is inferior, so the corresponding friction temperature of it is higher. Then, after that time, although the friction coefficient and power input of specimen S2 are lower than those of specimen S1, the temperature of specimen S2 is still higher than that of specimen S1 at the same test time. Because the surface temperature and mechanical property have strong impacts on the surface friction film, the differences in them between specimen S1 and S2 can induce different formation mechanisms of friction film, which would further affect the friction coefficient [18,19,20]. It is also found that at 250 km/h, temperature decline occurs in the test processes of two pressures for both specimen S1 and S2, as shown in Figure 7c,d, indicating that after the test process reaches a certain time (36 and 22 s indicated by red arrows for specimen S1 and S2 in Figure 7c, and 20 and 16 s indicated by red arrows for specimens S1 and S2 in Figure 7d, respectively), as the power input decreases continuously, the heat input on the surface is gradually lower than the heat output, so the temperature declines. It is also seen that the temperature of specimen S2 declines earlier than that of specimen S1 at each pressure, which is due to the fact that the temperature of specimen S2 is much higher than that of specimen S1. As is known, such a high temperature of specimen S2 can facilitate heat exchange, but the temperature decline rate of it is not fast because of its inferior thermal conductivity. However, at 100 km/h, no temperature decline occurs for both specimen S1 and S2 at each pressure (Figure 7a,b), which is mainly due to the fact that the heat output is not intense in such a short test time.

The friction coefficient of the surface is much influenced by the mechanical property and friction temperature, and they also have strong impacts on the formation of the surface friction film. As shown in Figure 7, there are great differences between the two specimens in the variation of the instantaneous friction coefficient under the four test conditions. At 100 km/h, the friction coefficient of specimen S1 at each pressure shows an uptrend. However, when the speed is up to 250 km/h, at each pressure, the friction coefficient declines obviously after the test process reaches a certain time (26 and 18 s indicated by blue arrow in Figure 7c,d, respectively). Due to the higher hardness and lower surface temperature, the surface deformation capacity of specimen S1 is much lower than specimen S2, which induces the contact and meshing of the friction pair to be more difficult in tests. The molecular mechanical friction theory can help to analyze the reasons for the increase of the friction coefficient. According to this theory, the friction coefficient, µ, can be expressed as:

(1) $$\mu =\beta +\alpha A_{r}/P$$

where β and α are the coefficients related to the mechanical meshing degree and molecular attraction of the surface, respectively. A_r_ is the real contact area and P is the pressure. As shown in the formula, it is seen that the increase of the real contact area and meshing degree of the friction pair is helpful to increase the friction coefficient. As the test time goes on, the contact area, meshing degree, and adhesion of friction pair are gradually increasing, so the friction coefficient of specimen S1 increases [19,25], as shown in Figure 7a–d. When the speed is increased from 100 to 250 km/h, due to the longer test time and higher surface temperature caused by the higher speed, the friction film gradually forms on the surface. The film can isolate the friction pair; that is, it exhibits a lubrication effect, so when the time reaches a certain moment, the friction coefficient no longer increases but decreases obviously, as shown in Figure 7c,d. Then, the film may be destroyed by the surface forces, inducing the friction coefficient increases again. For specimen S1, the low deformation capacity and good bonding between the graphite and matrix are unfavorable for the transfer of graphite particles to the friction surface [14], so the friction film forming at this speed may consist of a small amount of graphite, but more oxides [12,26]. The study on the friction film of specimen S1 will be continued based on the analysis of surface wear morphology and elements distribution in Section 3.4.

However, for specimen S2, at 100 km/h, the instantaneous friction coefficient generally shows a downtrend (Figure 7a,b). However, at 250 km/h, only after a certain time (30 and 40 s indicated by blue dash arrow at 0.4 and 0.8 MPa, respectively) at each pressure, the friction coefficient decreases continuously. Before that time, there are some fluctuations in it (Figure 7c,d). Compared with specimen S1, due to the lower hardness and higher surface temperature, the deformation capacity of specimen S2 is much enhanced during the test under each condition, inducing the contact and meshing of the friction pair to be easier [25], so the friction coefficient of specimen S2 is higher than that of specimen S1 within a period of time. Furthermore, the higher deformation capacity and inferior bonding between the graphite and matrix of specimen S2 are favorable for the transfer of graphite particles to the surface; that is, the graphite particles are more easily extruded to the surface to form the friction film, but the film is also easily destroyed because of the poor bonding between the graphite and matrix [12,13,14]. At 100 km/h and 0.4 MPa (Figure 7a), the decrease of the friction coefficient with time is due to the lubrication effect of graphite extruded. At the same speed, when the pressure is up to 0.8 MPa (Figure 7b), the downtrend of the friction coefficient is not obvious, which is due to the shorter testing time and higher surface force. As a result, the formation of the friction film is difficult and the destruction of it is easy [19]. When the speed is up to 250 km/h, the variations of the friction coefficient at two pressures are more complex (Figure 7c,d). Due to the higher surface temperature and deformation capacity at this speed, the graphite particles can transfer to the surface more easily. Therefore, within a stage since the beginning of the test, there is a decrease in the friction coefficient at each pressure. Then, in the following stage, due to the fact that the speed is still in a high range, the friction film of graphite can be destroyed under forces, which results in the increase of the friction coefficient. Then, after a stage of slight fluctuation of the friction coefficient, when the speed decreases to a certain value in the late test stage, the friction coefficient keeps decreasing, indicating that the destruction of graphite friction films is reduced when the speed decreases to a certain range [7,13,14,27]. It can also be found that when the speed at each pressure decreases to a value below 80 km/h, the friction coefficient keeps decreasing, as indicated by the olive arrows in Figure 7c,d. The study on the friction film of specimen S2 will also be continued based on the analysis of surface wear morphology and elements distribution in Section 3.4.

### 3.4. Wear Mechanisms under Different Conditions

**Figure 8:**
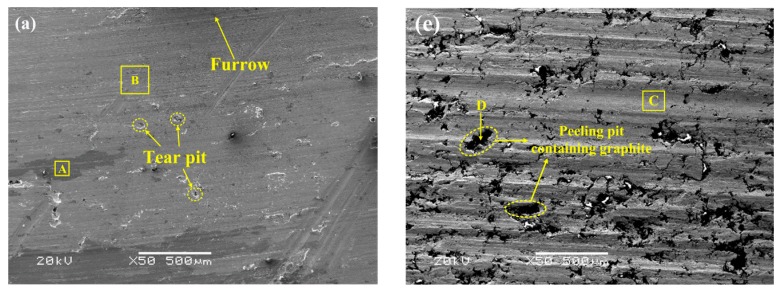

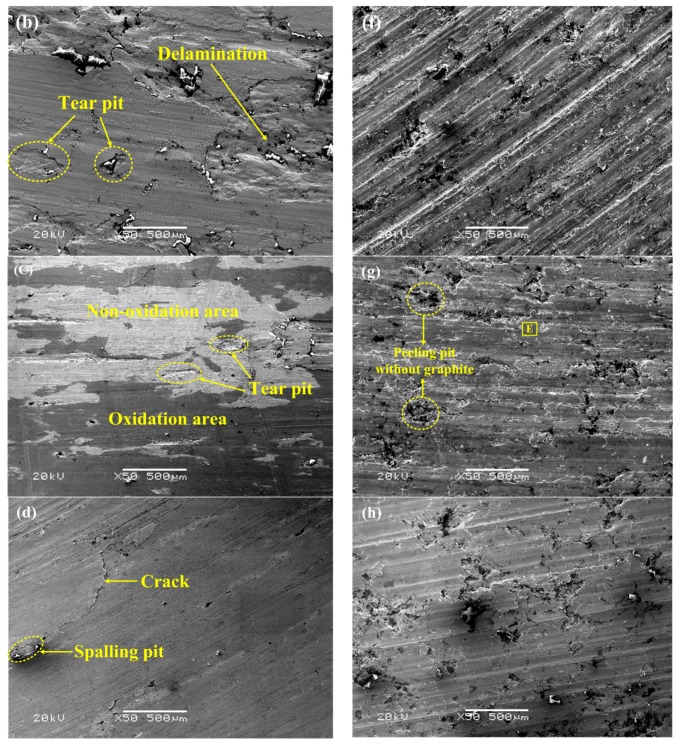
Surface morphologies after tests under different conditions: specimen S1 of (**a**) 100 km/h, 0.4 MPa; (**b**) 100 km/h, 0.8 MPa; (**c**) 250 km/h, 0.4 MPa; (**d**) 250 km/h, 0.8 MPa; specimen S2 of (**e**) 100 km/h, 0.4 MPa; (**f**) 100 km/h, 0.8 MPa; (**g**) 250 km/h, 0.4 MPa; (**h**) 250 km/h, 0.8 MPa.

Firstly, based on the analysis of the friction surface morphologies of the two specimens, the wear mechanisms under different conditions were studied. As shown in Figure 8, the surface morphologies of specimen S1 and S2 show great differences. Although there are pits on the surface for both specimen S1 and S2, the differences among the pits are obvious. Table 4 shows the EDS analysis results of area A, B, C, D, and E in Figure 8.

For specimen S1, at 100 km/h and 0.4 MPa, it is seen in Figure 8a that there are only slight furrows on the surface, indicating that the abrasive wear is not serious. Under this condition, only small oxidation areas appear on the surface, such as area A. This area shows a darker color compared with other areas (area B). In addition, a few small tear pits appear in the non-oxidation areas of the surface, indicating that slight adhesive wear occurs. At the same speed, increasing the pressure to 0.8 MPa, compared with Figure 8a, many deeper furrows and larger tear pits are found on the surface, and the pits show the obvious feature of unevenness inside and being torn, as shown in Figure 8b. It is indicated that serious abrasive and adhesive wear have occurred under the higher pressure and temperature of this condition [28,29]. Furthermore, delamination has also been found on the surface, because of the combined effects of a high force and strong adhesion. Under this condition, no obvious oxidation is found. Increasing the speed to 250 km/h, as shown in Figure 8c,d, it is evident that the oxidation effects have been greatly enhanced on surfaces at the two pressures, and the furrows caused by abrasion are also slight. Compared with 100 km/h, large dark oxidation areas can be found on the surfaces, indicating that the oxide films have formed. This is because the higher surface temperatures and longer test times at this speed promote the formation of oxide [26]. At 0.4 MPa, as is shown in Figure 8c, there are still some tear pits caused by adhesion in the non-oxidation areas, but in the oxidation area, no obvious pits arise. It is indicated that the oxide films can effectively isolate the friction pair to avoid adhesion, which reduces the material wear [30]. When the pressure is 0.8 MPa, as shown in Figure 8d, it is seen that the oxidation areas are so large that non-oxidation areas have been greatly reduced on the surface, so the adhesion wear of material has also been greatly reduced. However, in the oxidation areas, another type of pit appears. This is caused by the spalling of the oxide films [31,32]. The spalling pit is different from the tear pit caused by adhesion, and it shows the feature of smoothness inside without tearing, suggesting a certain degree of brittleness. In addition, another significant difference between the two types of pits is that there are some cracks around the spalling pits. The formation of the spalling pits is closely related to the cracks. When the brittle oxide film is subjected to external forces, the crack may initiate at certain parts of the film, such as the stress concentrating parts. Then, the crack propagates rapidly, causing the oxide film to be spalled; that is, oxidation wear occurs [33].

Compared with specimen S1, the wear of specimen S2 is obviously increased under the four same conditions, as shown in Figure 8e–h. In addition to some furrows, there are also many pits on the surfaces, but most of them show features different from the pits mentioned above. Due to the lower mechanical property of specimen S2, when the surface is subjected to external forces, the delamination of material can easily occur, which causes the material to be peeled off from the matrix. Meanwhile, the pits have formed. At 100 km/h and 0.4 MPa, as shown in Figure 8e, graphite is found in most of the pits, indicating that the pits easily form at the location where graphite particles are found, which is due to the low mechanical property of graphite [34]. For specimen S2, the poor interfacial bonding between the graphite and matrix may cause the graphite particles to be detached from the matrix under the force, but under this condition, due to the low pressure and short test time, many graphite particles in the pits have not been detached. Meanwhile, friction films have not formed effectively, such as area C (Figure 8e), and the content of Cu is still high in the area due to a lack of covering by the films. So, under this condition, both the friction coefficient and material wear are high (Figure 6). At the same speed, increasing the pressure to 0.8 MPa, compared with the pressure of 0.4 MPa, the higher pressure and temperature can cause the deformation more easily, so more furrows with a greater depth have formed on the surface (Figure 8f). Furthermore, due to the short test time (about 9 s) at this pressure, the number of pits containing graphite on the surface is obviously smaller, and the wear of material is also high. When the speed is up to 250 km/h, it is evident that there are still many furrows and pits on the surfaces of the two pressures, and it is obvious that some graphite particles in the pits have been detached from the matrix, as shown in Figure 8g,h. Under the pressure applied, the detached graphite particles, together with other debris, can be flattened on the surface, so they can play the role of the isolating friction pair [13]. An examples of this is area E (Figure 8g), compared with area C (Figure 8e), for which it can be found that the content of carbon in the area has increased; meanwhile, the content of Cu decreases, indicating that the lubricating friction films have formed. However, the friction films under this condition show low continuity [14], and they are inclined to be peeled off. Thus, compared with specimen S1, the lubricating effect of the friction films of specimen S2 is lower, causing the higher wear rate (Figure 6b).

**Figure 9:**
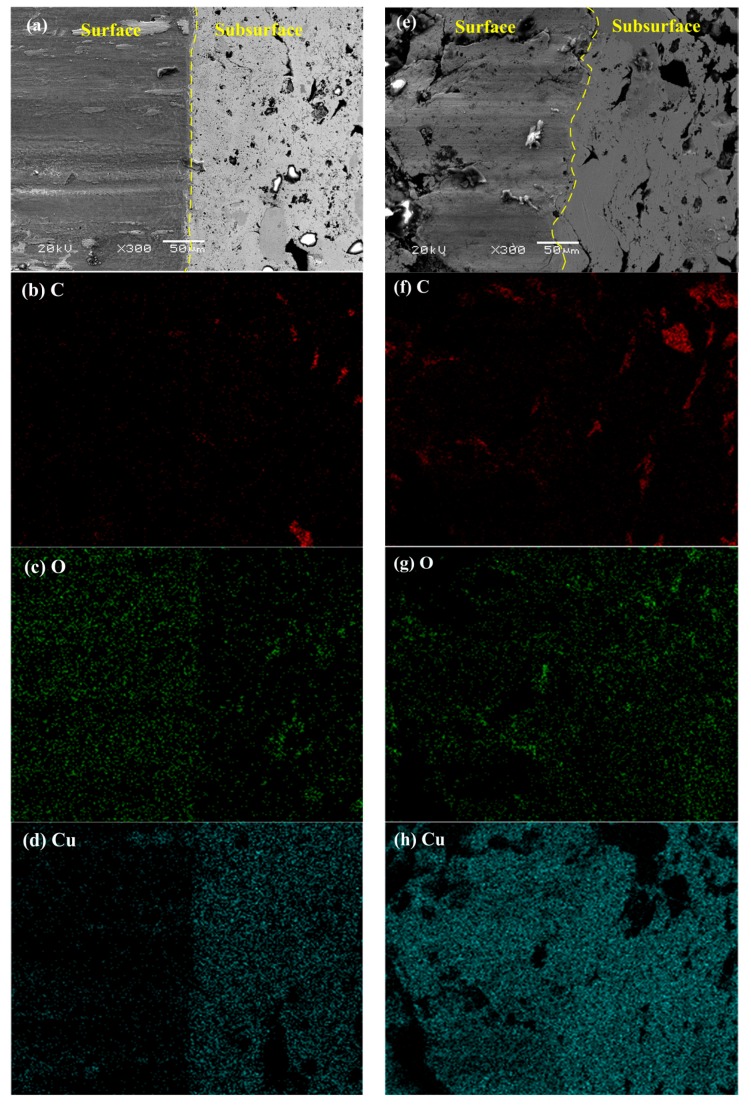
Morphologies and major elements distribution on the friction surface and subsurface of inclined-plane samples at 250 km/h and 0.4 Mpa for specimen S1 of (**a**–**d**) and specimen S2 of (**e**–**h**): (**a**,**e**) morphologies; (**b**,**f**) distribution of C; (**c**,**g**) distribution of O; (**d**,**h**) distribution of Cu.

Based on the analysis of morphologies and elements distribution of inclined-plane samples, the differences of friction films on the surface of specimen S1 and S2 were further studied. Figure 9 shows the morphologies and major elements distribution of the surface and subsurface of the two specimens at 250 km/h and 0.4 MPa. As shown in Figure 9, compared with specimen S2, the surface wear of specimen S1 is lower, and the boundary between the surface and subsurface is much straighter due to its smaller deformation (Figure 9a), while the deformation near the boundary in the subsurface of specimen S2 is very large (Figure 9e).

For specimen S1, it is seen that the difference between the surface and subsurface in the distribution of C is small (Figure 9b); however, the differences in the distribution of O and Cu are obvious (Figure 9c,d). This is due to the fact that the higher temperature under this condition can promote the formation of an oxide film [26], so the content of O on the surface is much higher than that on the subsurface. Meanwhile, the content of Cu on the surface significantly decreases by the covering of oxide films. For specimen S2, the distribution of C on the surface is more uniform than that on the subsurface (Figure 9f), which is because the large deformation and low interfacial bonding between the graphite and matrix can cause the graphite to transfer to the surface easily [13,14]. However, the distributions of O and Cu on the surface and subsurface display few differences (Figure 9g,h). Based on the analysis above, it can be concluded that under this condition, the friction films on the surface of specimen S2 are mainly composed of graphite, while the content of oxide in the films is low; but for specimen S1, the friction films are mainly composed of oxide and a little amount of graphite. For both the two specimens, the high speed favors the formation of the friction film on the surface. In addition, when the Cu-coated graphite is used in the materials, since it is difficult for the graphite particles to transfer to the surface, in order that the graphite can play a better role in lubrication, it may be efficient to increase the content of graphite in the material; that is, more graphite may transfer to the friction surface and be involved in the formation of friction films.

### 3.5. Phase Composition

**Figure 10:**
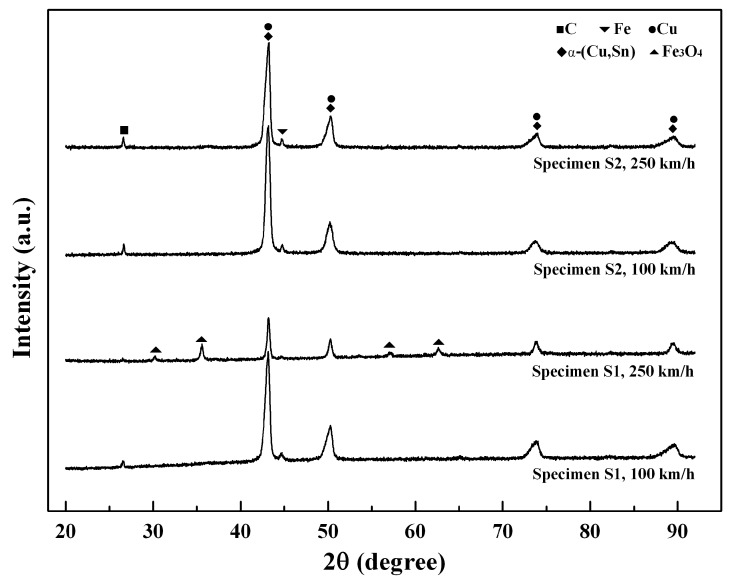
XRD analysis results of friction surfaces for two specimens at two speeds and 0.4 MPa.

After the friction and wear tests, we performed XRD analysis for the two specimens to test the phase composition on the surfaces. The analysis results of the friction surfaces at 0.4 MPa and two speeds are shown in Figure 10. For specimen S1, compared with the speed of 100 km/h, the diffraction peak of Fe_3_O_4_ obviously arises at 250 km/h, indicating that the oxide friction films have formed on the surface at this high speed. Due to the isolation effect of the oxide films on the friction pair, the diffraction intensity of Cu at 250 km is much lower than that at 100 km/h. However, for specimen S2, the diffraction peak of oxide is not found at each speed, but the diffraction peak of C is obviously stronger than that of specimen S1, indicating that the content of graphite in friction films of specimen S1 is higher, especially at the speed of 250 km/h, and the diffraction intensity of Cu at 250 km is also lower than that at 100 km/h for specimen S1, which is due to the fact that the friction films can easily form at a high speed. The above analysis shows that the increase of speed is favorable to the formation of the friction film on the surface for both of the specimens.

## 4. Conclusions

Based on the investigation of the tribological performance of copper-based powder metallurgical friction material with the lubricant of Cu-coated or uncoated graphite particles, the following conclusions can be drawn:

1. Under the same content of graphite, with the lubricant of Cu-coated graphite, the hardness, relative density, thermal conductivity, and interfacial bonding between the graphite and matrix of the copper-based powder metallurgical material can be greatly improved.
2. Under the same test condition, the average friction coefficient and wear rate of specimen S1 are both lower than those of specimen S2. The higher friction coefficient of specimen S2 is beneficial for braking, but it can also increase the wear of material.
3. Under a low initial test speed, the variations of the instantaneous friction coefficient for specimen S1 at two pressures both show an uptrend, but the variations of it for specimen S2 at two pressures almost show a downtrend. Under a high initial test speed, when the braking process reaches a certain stage, the decrease in the instantaneous friction coefficient occurs obviously in specimen S1 at each pressure, and the friction coefficient then increases again. For specimen S2 at each pressure, the instantaneous friction coefficient firstly decreases, and then after a stage of fluctuation, it keeps decreasing to the end.
4. The friction temperature of specimen S1 at each condition is much lower than that of specimen S2. At a low initial test speed, for the two specimens at each pressure, the friction temperature increases with time. At a high initial test speed, when the braking process reaches a certain stage, the temperature decline arises in both of the two specimens at each pressure.
5. Due to the great delamination effect on the surface of specimen S2, the wear of it is more serious than that of specimen S1. A high speed is favorable to the formation of friction films on the surface for the two specimens. The friction films of specimen S1 contain more graphite and less oxide, and show low continuity; however, the friction films of specimen S1 mainly consist of oxide and show high continuity.

## Figures and Tables

**Table 1:** Chemical composition and properties of the two specimens.

Material Type	Content of Components (wt.%)						Hardness (HB)	Relative Density (%)	Thermal Conductivity Coefficient (W/mK)
	Cu	Fe	SiO_2_	Sn	Cu-Coated Graphite	Uncoated Graphite			
Specimen S1	74	10	4	4	8	0	93	98.8	119.1
Specimen S2	78	10	4	4	0	4	70	91.6	70.3

**Table 2:** Test groups and results of friction and wear test.

Group Number	Specimens	P (MPa)	v_0_ (km/h)	Braking Time (s)	Average Friction Coefficient	Wear Rate (mg/kJ)	Maximum Friction Temperature (°C)
1	S1	0.4	100	17.4	0.34	1.10	128.7
2	S1	0.4	150	25.3	0.31	0.81	
3	S1	0.4	200	34.2	0.30	0.74	
4	S1	0.4	250	42.6	0.29	0.71	303.1
5	S1	0.8	100	9.6	0.39	9.23	156.1
6	S1	0.8	150	15.5	0.33	3.52	
7	S1	0.8	200	24.1	0.27	1.01	
8	S1	0.8	250	29.9	0.26	0.12	342.6
9	S2	0.4	100	15.5	0.36	25.20	182.2
10	S2	0.4	150	24.3	0.35	17.21	
11	S2	0.4	200	32.5	0.33	13.51	
12	S2	0.4	250	41.9	0.30	8.92	411.6
13	S2	0.8	100	8.3	0.41	29.40	211.4
14	S2	0.8	150	13.5	0.37	22.81	
15	S2	0.8	200	18.5	0.34	17.64	
16	S2	0.8	250	29.0	0.28	9.54	557.5

**Table 3:** EDS analysis results of area A, B, C, and D in Figure 5a.

Area	Content of Elements (wt.%)					
	C	O	Si	Sn	Fe	Cu
A	5.65	0.82	0	4.89	1.23	87.41
B	0	0	0	0	100	0
C	95.43	0.99	0.33	1.11	0.57	1.57
D	4.74	48.84	44.66	0	0.92	0.84

**Table 4:** EDS analysis results of area A, B, C, D, and E in Figure 8.

Area	Content of Elements (wt.%)				
	C	O	Si	Fe	Cu
A	4.27	26.99	3.61	57.72	7.41
B	8.83	5.43	0.44	17.28	68.02
C	12.79	4.56	2.74	2.94	76.97
D	89.93	1.11	0.28	0.47	8.31
E	42.47	13.97	2.55	19.10	21.91

## Appendix A

1. Computation formula of test inertia:

(A1) $$I=10^{-6}\theta MA_{2}R_{1}{}^{2}R_{2}{}^{2}/4A_{1}R_{3}{}^{2}$$

where, I: Test inertia, kg m^2^; θ: Energy distribution coefficient; M: Train axle load, kg; A_1_: Disk friction area of train, cm^2^; A_2_: Test friction area, cm^2^; R_1_: Train wheel radius, mm; R_2_: Test friction radius, mm; and R_3_: Train friction radius, mm.

2. Computation formula of instantaneous friction coefficient:

(A2) $$\mu =10^{3}T/RN$$

where, μ: instantaneous friction coefficient; T: Friction torque, Nm; R: Test friction radius, mm; and N: Test load, N.

3. Computation formula of wear rate:

(A3) $$a=2\times 10^{6}(m_{1}-m_{2})/I\omega^{2}$$

where, a: Wear rate, mg/kJ; m_1_: Sample mass before test, g; m_2_: Sample mass after test, g; I: Test inertia, kg m^2^; and ω: Test angular velocity, rad/s.

4. Computation formula of instantaneous power input:

(A4) P=μpv

where, P: Instantaneous power input, MPa km/h; μ: instantaneous friction coefficient; p: Instantaneous test pressure, MPa; and v: Instantaneous friction speed, km/h.
